# Examining Visual Attention to Tobacco Marketing Materials Among Young Adult Smokers: Protocol for a Remote Webcam-Based Eye-Tracking Experiment

**DOI:** 10.2196/43512

**Published:** 2023-04-13

**Authors:** Julia Chen-Sankey, Maryam Elhabashy, Stefanie Gratale, Jason Geller, Melissa Mercincavage, Andrew A Strasser, Cristine D Delnevo, Michelle Jeong, Olivia A Wackowski

**Affiliations:** 1 Center for Tobacco Studies School of Public Health Rutgers Biomedical and Health Sciences New Brunswick, NJ United States; 2 Center for Tobacco Studies Rutgers Biomedical and Health Sciences New Brunswick, NJ United States; 3 Department of Psychology Princeton University Princeton, NJ United States; 4 School of Medicine University of Pennsylvania Philadelphia, NJ United States

**Keywords:** eye tracking, remote eye tracking, e-cigarette marketing, young adults, mobile phone

## Abstract

**Background:**

Eye tracking provides an objective way to measure attention, which can advance researchers’ and policy makers’ understanding of tobacco marketing influences. The development of remote webcam-based eye-tracking technology, integrated with web-based crowdsourcing studies, may be a cost-effective and time-efficient alternative to laboratory-based eye-tracking methods. However, research is needed to evaluate the utility of remote eye-tracking methods.

**Objective:**

This study aimed to detail the process of designing a remote webcam-based eye-tracking experiment and provide data on associations between participant characteristics and the outcomes of experiment completion.

**Methods:**

A total of 2023 young adult (aged 18-34 years) cigarette smokers in the United States were recruited to complete a web-based survey that included a 90-second remote eye-tracking experiment that examined attention to e-cigarette marketing materials. Primary outcome measures assessed the completion of the remote eye-tracking experiment—specifically, experiment initiated versus not initiated, experiment completed versus not completed, and usable versus nonusable eye-tracking data generated. Multivariable logistic regressions examined the associations between outcome measures and participants’ sociodemographic backgrounds, tobacco use history, and electronic devices (mobile vs desktop) used during the experiment.

**Results:**

Study recruitment began on April 14, 2022, and ended on May 3, 2022. Of the 2023 survey participants, 1887 (93.28%) initiated the experiment, and 777 (38.41%) completed the experiment. Of the 777 participants who completed the experiment, 381 (49%) generated usable data. Results from the full regression models show that non-Hispanic Black participants (adjusted odds ratio [AOR] 0.64, 95% CI 0.45-0.91) were less likely to complete the eye-tracking experiment than non-Hispanic White participants. In addition, female (vs male) participants (AOR 1.46, 95% CI 1.01-2.11), those currently using (vs not using) e-cigarettes (AOR 2.08, 95% CI 1.13-3.82), and those who used mobile (vs desktop) devices (AOR 5.10, 95% CI 3.05-8.52) were more likely to generate usable eye-tracking data.

**Conclusions:**

Young adult participants were willing to try remote eye-tracking technology, and nearly half of those who completed the experiment generated usable eye-tracking data (381/777, 49%). Thus, we believe that the use of remote eye-tracking tools, integrated with crowdsourcing recruitment, can be a useful approach for the tobacco regulatory science research community to collect high-quality, large-scale eye-tracking data in a timely fashion and thereby address research questions related to the ever-evolving tobacco marketing landscape. It would be useful to investigate techniques to enhance completion rates and data usability.

**International Registered Report Identifier (IRRID):**

RR1-10.2196/43512

## Introduction

### Traditional Eye-Tracking Tools and Emergent Challenges

Eye tracking has become an increasingly popular method of assessing visual engagement with marketing and communication materials in tobacco control research and tobacco regulatory science [1,2]. In particular, eye tracking has been used to gain insight into how people pay attention to pro- or antitobacco communication efforts, such as industry marketing tactics [3,4], warning labels [5-10], and other tobacco-related health messages [11-13]. Unlike self-reported surveys or qualitative assessments that could introduce recall and response biases, eye tracking provides objective measures of visual attention to tobacco marketing stimuli that are predictive of information recall and processing [1,14], which can lead to changes in perceptions about tobacco products. Studies using eye tracking can help inform researchers and policy makers about the influence of tobacco marketing exposure among various populations.

Eye tracking is typically conducted in an in-person research setting, with participants entering a computer laboratory equipped with specialized, infrared eye-tracking equipment, operated by at least 1 research staff member. Challenges associated with traditional eye-tracking studies include the costly and tedious process of study implementation [15], which requires bringing people into a laboratory where they can be tracked individually, as well as geographic restrictions regarding the sample because most study recruitment takes place locally. Such challenges have been exacerbated by the recent COVID-19 pandemic, further highlighting the need for remote, web-based alternatives.

### Advantages of Remote Webcam-Based Eye Tracking

Recent developments in web-based remote eye tracking, which accesses users’ webcams to capture video images of the face to predict gaze location on the screen, as opposed to the traditional corneal and pupil reflection used in most laboratory eye trackers, make this approach an attractive, available alternative when laboratory studies are not possible. This approach may largely remove the restrictions of conducting traditional eye-tracking studies [16]. There are several benefits of using remote eye-tracking studies. Mainly, these studies can support large-scale, crowdsourced web-based research without geographic restrictions or restrictions pertaining to equipment or staff availability; researchers are also able to immediately and simultaneously obtain eye-tracking data from a large sample of participants. In addition, participants can perform tasks whenever and wherever they choose, as long as they have a computer or smartphone or tablet device equipped with a camera and an internet connection. The ability to conduct an eye-tracking study on participants’ own devices in their natural environment makes it ideal for studies that examine participants’ attention to commercial marketing materials seen in real life rather than within a controlled laboratory environment.

Remote eye tracking may be especially useful for conducting tobacco marketing research. First, compared with a laboratory setting, performing eye-tracking tasks on personal electronic devices in participants’ natural environment may more realistically mimic the context of tobacco marketing exposure, particularly when the marketing stimuli consist of materials publicized on websites and social media platforms. As social media and web-based content become an increasingly common channel of tobacco marketing exposure for young people [17-20], the ability to perform eye-tracking tasks on personal electronic devices becomes especially important. In addition, the capability of remote eye-tracking technology to be compatibly used with large-sample crowdsourced data will facilitate researchers’ and regulators’ understanding of the influence of tobacco marketing materials at the population level and among subgroups (eg, tobacco users, former users, and never users). Finally, studies examining ever-changing tobacco marketing practices require designs that reflect the most recent marketing tactics and policy changes. Research that integrates remote eye-tracking technology with crowdsourcing data collection can provide rapid responses to surveillance needs and regulatory challenges requiring immediate attention.

Recent research studying the efficacy of remote eye-tracking technology shows that this new technology produces satisfactory accuracy (defined as the capacity to truthfully describe the location of a person’s gaze on a screen) compared with the data gathered from a traditional infrared-based eye tracker used in a controlled laboratory setting [16,21-24]; for example, researchers were able to obtain comparable results from a remote versus laboratory-based eye-tracking experiment in 3 tasks commonly used for eye tracking (fixation, pursuit, and free viewing) and found that webcam-based eye tracking is generally suitable for all 3 tasks and holds promise in cognitive science–based research [21]. Together with the potential aforementioned benefits, remote eye tracking may be a potentially viable alternative for researchers that substantially reduces the time and costs associated with conducting a laboratory-based eye-tracking study.

### Research Questions and Study Objectives

**Figure 1:**
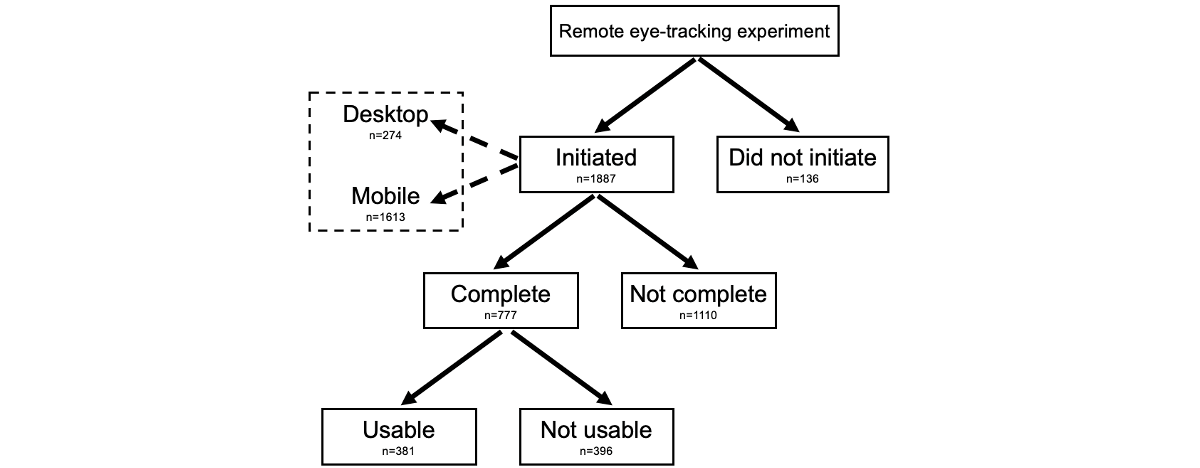
Remote eye-tracking experiment flowchart and distribution of experiment completion. Definition of initiating the experiment: participants who chose to proceed to the eye-tracking experiment using either desktop or mobile devices. Definition of complete: participants who completed the entire experiment (participants who started the experiment but did not meet technical requirements or closed the browser or had timed out before reaching the end were considered "not complete"). Definition of usable: participants whose gaze and emotion were trackable during their session, that is, had proper lighting and did not move.

Because of the novel nature of remote eye-tracking platforms, relatively few publications describe the process of configuring such experiments and integrating them with large-scale crowdsourcing survey platforms. In particular, there are questions of how this innovative method can be used to examine the influence of tobacco marketing materials among a large sample of tobacco users and which subpopulations (characterized by age, biological sex, race and ethnicity, and other factors) may be more likely to participate in remote eye-tracking studies. Using a large sample of young adult cigarette smokers who participated in a web-based survey through crowdsourcing, this study had two goals: (1) to provide a detailed description of our approach to configuring a remote eye-tracking experiment embedded in Qualtrics (Qualtrics International Inc), a research company used for conducting behavioral science research, including tobacco research and (2) to document associations with demographic, smoking history, and other characteristics of study participants who completed various stages of the remote eye-tracking experiment (Figure 1). Given the potential utility of remote eye-tracking tools for academic researchers, this paper further describes key features of the platform and integrated survey system for remote eye-tracking studies, details potential challenges that may occur before and during data collection, and recommends solutions to streamline the use of the tool and improve data collection quality.

## Methods

### Remote Eye-Tracking System

**Figure 2:**
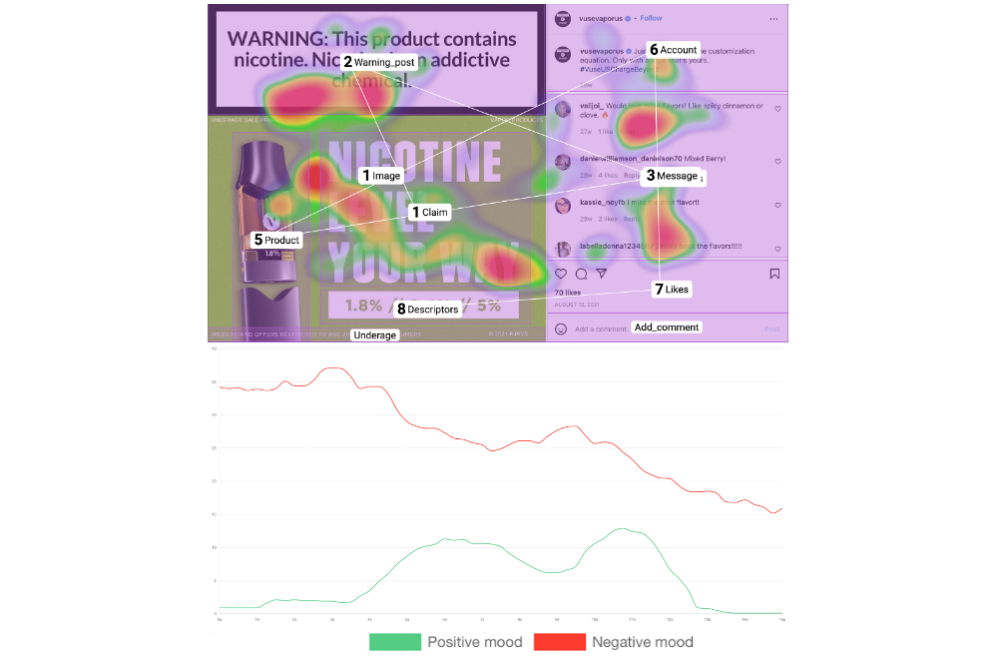
Example of gaze metrics generated by remote eye tracking.

This research used Sticky by Tobii (Tobii AB), a remote webcam-based eye-tracking tool that provides a cloud-based eye-tracking measurement of attention and emotional responses. This tool combines survey questions with remote webcam eye-tracking technology that can be used with cameras for tablet devices and mobile phones as well as webcams for laptop and desktop computers. It can be integrated with a web-based survey tool such as Qualtrics, which can help expand the research team’s access to a larger study sample while providing various options for survey formatting. Through designating certain areas of interest (AOIs) on the experimental stimuli, researchers can identify participants’ attention and emotional responses to different areas of the stimuli. Data generated from the experiment are automatically streamed to the cloud as they are recorded. On the basis of the analysis of participants’ webcam video recordings, the cloud-based eye-tracking software platform generates attention data (measured by gaze patterns via webcam-based eye tracking) and emotion metrics (measured by various emotional expressions via webcam-based facial coding) within hours of data collection (refer to Figure 2 for examples of gaze patterns and emotion metrics). The software also generates heat maps of static gaze visualizations at individual and group levels, which serve as a graphical representation of fixation counts and duration. In addition, researchers can insert questionnaires before or after the stimuli presentation. Data collected from the remote eye-tracking tool and the integrated web-based survey platform can be downloaded from the relevant platform and merged using unique participant IDs.

### Study Participants and Procedures

Between April 14, 2022, and May 3, 2022, we recruited a web-based convenience sample (N=2023) of young adult cigarette smokers (aged 18-34 years) via Qualtrics. Qualtrics recruits participants through their web-based study panels by sending email invitations to potentially eligible participants. Participants interested in the study first completed a screening survey to confirm their eligibility, for which the criteria were as follows: (1) aged between 18 and 34 years, (2) smoked at least 1 cigarette in the past 30 days, and (3) smoked ≥100 cigarettes over their lifetime. Eligible participants were then shown an informed consent page that described the purpose of the study (to examine the influence of e-cigarette marketing features) and provided details about the survey and the embedded remote eye-tracking experiment. They were instructed that they would need to turn on their cameras to complete the eye-tracking experiment. Participants were also told that their faces would be recorded by video during the remote eye-tracking task; however, they were assured that the researchers would not have access to any recordings of their faces or surroundings. In addition, participants were told that they would receive a complete code for full survey compensation even if they decided to skip the remote eye-tracking section of the study.

Participants who agreed with the informed consent proceeded to the web-based survey, where they answered a series of questions about their sociodemographic background (eg, biological sex, race and ethnicity, and subjective financial status) and tobacco use history (eg, current cigarette smoking and e-cigarette use frequency). Next, participants proceeded to the introduction of the remote eye-tracking experiment, which stated, “Now, we will ask you to look at a few e-cigarette images through a remote eye-tracking experiment. Your participation in this experiment is very important as your response will help us understand the impactful features of e-cigarette advertisements. We greatly appreciate your time and efforts in completing this experiment.” Subsequently, participants were asked to indicate the device they were using or opt to skip the eye-tracking section: “If you wish to proceed, please select the type of device you are currently using for completing the survey,” with the response options “I do not wish to proceed,” “Desktop or laptop screen,” “Mobile, tablet,” and “Mobile, phone.” Those who chose the first option were directed to the end of the survey, where they viewed health education messages about the harms of tobacco use and methods of quitting tobacco before they exited the survey.

As the software requires different experiment setups for mobile (tablet and phone) and desktop (desktop and laptop) devices, participants who wished to initiate the experiment were directed to the corresponding mobile- or desktop-based experiment in accordance with the device they chose. The total time participants engaged with the entire course of the experiment was approximately 5 minutes, which included reading the instructions, processing calibrations, viewing the experimental stimuli, and completing the survey items. Participants who completed the experiment were then directed to view the health education messages on Qualtrics before exiting the survey. In the following sections, we describe the process of configuring the remote eye-tracking experiment and embedding it within the Qualtrics web-based survey platform.

### Remote Eye-Tracking Experiment Setup

#### Experiment Setup and Calibration

**Figure 3:**
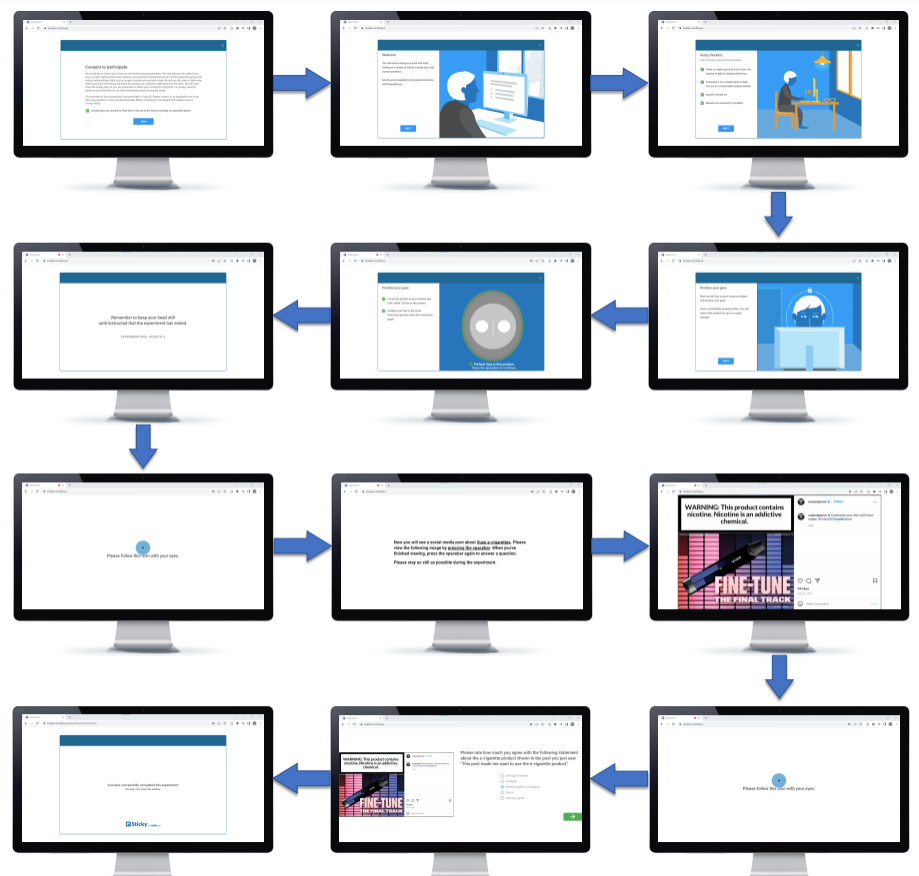
Remote eye-tracking platform use instructions (screenshots).

After choosing to initiate the remote eye-tracking experiment, participants were shown instructions related to the use of the software (Figure 3). After they completed reading through a brief consent message, they were told that their cameras needed to be activated to detect and properly position their gaze before they viewed the experiment’s stimuli. After the default setup for calibration had been set up, 2 calibration points were used in our experiment. The first calibration point was set up before participants saw the instructions to properly view the stimuli, and the second calibration point was set up after participants had viewed the stimuli. During calibration, participants were told to attentively follow a moving icon on the screen.

After completing the first calibration, participants were presented with instructions specific to the experiment, which differed slightly for mobile- and desktop-based experiments. For the mobile-based experiment, the instructions read, “Now you will see a social media post about e-cigarettes. Please view the following image by tapping on your screen. When you are finished viewing (scrolling is enabled), tap your screen again to answer the question. Please stay as still as possible during the experiment.” The instructions for the desktop-based experiment differed slightly in that scrolling was disabled, and participants could click the mouse or press the spacebar to proceed. We set a duration (ie, 20 seconds) for presenting the instructions, enabling the software to proceed automatically if a participant did not advance manually.

#### Designing Stimuli for the Eye-Tracking Experiment

**Figure 4:**
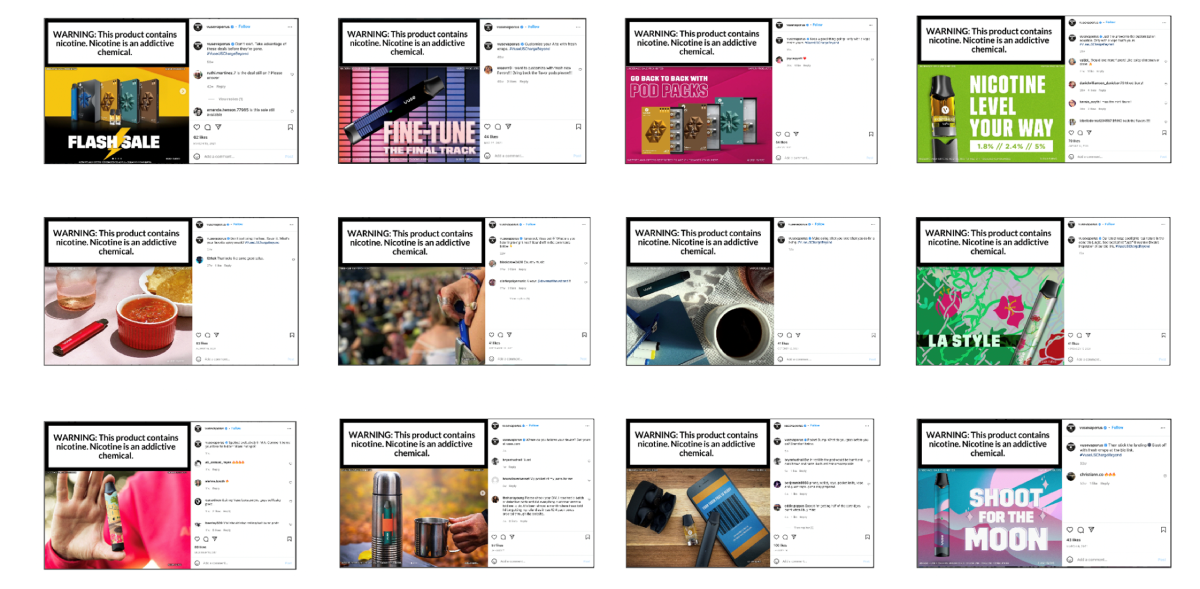
Vuse social media posts used in the experiment (screenshots).

After advancing, participants viewed the experiment’s stimuli that consisted of static images of 12 e-cigarette social media posts obtained directly from the official Instagram account of Vuse, a commonly used e-cigarette brand (Figure 4). To fit the stimuli for each experiment on the screen, the posts used in the mobile-based experiment were directly obtained from Instagram’s mobile app, whereas the same posts used in the desktop-based experiment were obtained from Instagram’s website. For both experiments, we randomly assigned 1 of the 12 images to be presented to the participants by selecting the option “randomly displaying 1 out of 12 media” within the experiment setup page. We set each stimulus to *fit to screen* for the desktop-based experiment and be *scrollable* for the mobile-based experiment. Participants using desktop devices were allowed to proceed through all stimuli *on mouse click* or *by pressing the spacebar*, and those using mobile devices were allowed to proceed by tapping the screen. We also set a *maximum duration* (ie, 15 seconds) for presenting each stimulus, in the event that a study participant did not advance manually.

While preparing for the experiment, the research team created AOIs within each stimulus. The AOIs were based on systematic coding of the stimuli before designing the remote eye-tracking experiment. Two coders carefully viewed each stimulus and marked visible features (including the image portion and the comment section of the social media posts). To obtain a final set of codes for each stimulus, agreement on the coding was decided after discussion with a third coder. The research team set up the AOIs by creating either rectangular or free-form shapes around the features. We named each AOI according to its content (eg, warning label, package, and marketing claim), which was directly informed by the coding results. Participants were not able to view the boundaries or names of the AOIs.

#### Setting Up the Survey Question

Immediately after the e-cigarette stimuli presentation, participants were asked about their willingness to use e-cigarettes. After completing this survey question, participants were told that they had successfully completed the remote eye-tracking experiment and were automatically returned to the Qualtrics survey.

### Integration of the Remote Eye-Tracking Experiment With the Qualtrics Survey Platform

#### Setting Up the Audience

The audience portion included selecting the participant source, setting the panel size, and configuring participant settings. In selecting the participant source, we were given the option of sourcing our own participants or having Tobii recruit and field participants based on our eligibility criteria. Our research team chose the former option, and we recruited participants through Qualtrics’ web-based study panel.

#### Embedding the Remote Eye-Tracking Experiment With Qualtrics

As described previously, the Qualtrics survey for this study was configured to redirect users to the remote eye-tracking experiment depending on the device being used by the participant. Consequently, we were required to create 2 remote eye-tracking experiment blocks within our Qualtrics survey: one for desktop users and another for mobile users. We then used display logic on Qualtrics to route people to the relevant eye-tracking experiment. Configuring participant settings gave us the option to create a redirect link and a JavaScript link through which participants could access the eye-tracking experiment. Therefore, we configured participant settings using the Qualtrics survey platform by including the remote eye-tracking experiment *in an external survey platform with JavaScript*. Upon selecting Qualtrics as the survey provider, we were prompted to read a series of instructions on how to properly embed the remote eye-tracking experiment into the Qualtrics survey.

#### Verification, Review, and Launch

After setting up the audience and embedding eye-tracking experiments within the Qualtrics survey, we proceeded to the verification page to assess the functionality of our experiments before officially launching them. We ran multiple test sessions for both the mobile- and desktop-based experiments before the official launch. The experiment took <90 seconds to complete. After making a few changes, we launched the experiment, at which point adjustments could no longer be made. The crowdsourcing data collection through Qualtrics’ web-based panel took 20 days, and data were obtained from young adult cigarette smokers (N=2023) who completed the survey and passed Qualtrics’ data quality checks.

### Assessing the Characteristics of the Participants Who Completed the Remote Eye-Tracking Experiment and Generated Usable Data

#### Survey Measures

A series of survey measures programmed in the Qualtrics survey platform assessed participants’ characteristics and tobacco use history. These measures were used to assess which subgroups of young adult cigarette smokers would be more likely to initiate and complete the remote eye-tracking experiment. Specifically, sociodemographic characteristics included biological sex, age, race and ethnicity, sexual orientation, marital status, highest education level, current employment status, current school enrollment, and subjective financial status (Table 1). Tobacco use statuses included cigarette smoking frequency (daily vs nondaily) and current e-cigarette use status (current use vs nonuse). Finally, devices (mobile vs desktop) used for completing the survey and remote eye-tracking experiment were also included in the analysis.

**Table 1:** Characteristics of participants (web-based sample of young adult cigarette smokers) and remote eye-tracking experiment completion on a Qualtrics survey.

			Full sample (N=2023), n (%)		Remote eye-tracking experiment completion								*P* value	
					Did not initiate the experiment (n=136), n (%)^a^		Initiated but did not complete the experiment (n=1110), n (%)^a^		Completed the experiment but generated nonusable data (n=396), n (%)^a^	Completed the experiment and generated usable data (n=381), n (%)^a^				
**Biological sex^b^**														.02
	Female	1017 (50.3)		65 (6.4)		624 (61.4)		120 (11.8)			208 (20.5)			
	Male	1006 (49.7)		71 (7.1)		599 (59.6)		162 (16.1)			174 (17.2)			
**Age range (years)**														.14
	18-21	182 (9)		16 (8.8)		103 (56.6)		17 (9.3)			46 (25.3)			
	22-25	475 (23.5)		38 (8)		289 (60.8)		65 (13.6)			83 (17.6)			
	26-29	682 (33.7)		36 (5.3)		415 (60.9)		97 (14.2)			134 (19.5)			
	30-34	684 (33.8)		46 (6.7)		418 (61.1)		102 (14.9)			118 (17.3)			
**Race and ethnicity**														.07
	Non-Hispanic White	1284 (63.5)		78 (6.2)		770 (60)		188 (14.7)			248 (19.4)			
	Non-Hispanic Black	208 (10.3)		12 (5.7)		145 (69.9)		15 (7.2)			36 (17.2)			
	Hispanic	366 (18.1)		28 (7.6)		216 (59.1)		57 (15.5)			65 (17.7)			
	Non-Hispanic other^c^	165 (8.2)		16 (9.8)		97 (58.9)		20 (12.3)			32 (19)			
**Sexual orientation**														.86
	Heterosexual	1491 (73.7)		97 (6.5)		908 (60.9)		209 (14)			277 (18.6)			
	Other orientations^d^	532 (26.3)		39 (7.3)		317 (59.6)		72 (13.5)			104 (19.6)			
**Marital status**														.003
	Married	431 (21.3)		36 (8.4)		269 (62.4)		69 (16.1)			57 (13.3)			
	Living with partner	490 (24.2)		27 (5.5)		268 (54.7)		74 (15.1)			121 (24.7)			
	Single, never married	1001 (49.5)		67 (6.7)		629 (62.8)		126 (12.6)			179 (17.9)			
	Other^e^	101 (5)		6 (5.9)		59 (58.8)		12 (11.8)			24 (23.5)			
**Highest education level**														.15
	Less than high school	139 (6.9)		10 (7.5)		82 (59)		18 (13)			29 (21.6)			
	High school	791 (39.1)		47 (6)		483 (61)		111 (14.1)			150 (19)			
	Less than college degree	724 (35.8)		50 (6.9)		448 (61.9)		84 (11.6)			142 (19.6)			
	College degree or higher	368 (18.2)		31 (8.1)		222 (57.6)		71 (18.4)			62 (16)			
**Current employment status**														.12
	Work full time	1131 (55.9)		86 (7.6)		694 (61.4)		161 (14.2)			190 (16.9)			
	Work part time	299 (14.8)		14 (4.7)		189 (63.2)		38 (12.7)			58 (19.4)			
	Unemployed	375 (18.5)		24 (6.4)		217 (57.9)		57 (15.2)			77 (20.5)			
	Other^f^	218 (10.8)		12 (5.5)		125 (57.3)		26 (11.9)			55 (25.2)			
**Currently in school**														.99
	No	1541 (76.2)		103(6.7)		934(60.6)		214(13.9)			290(18.8)			
	Yes	482 (23.8)		33(6.9)		291(60.5)		66(13.7)			92(18.9)			
**Subjective financial status**														.67
	Comfortable	563 (27.8)		37 (6.6)		353 (62.7)		76 (13.5)			97 (17.2)			
	Meet needs	544 (26.9)		45 (8.1)		318 (58.3)		72 (13.1)			109 (20)			
	Just meet needs	611 (30.2)		36 (5.9)		363 (59.5)		87 (14.3)			125 (20.3)			
	Do not meet needs	305 (15.1)		19 (6.2)		188 (61.7)		47 (15.4)			51 (16.7)			
**Smoked cigarettes**														.18
	Daily	744 (36.8)		43 (5.8)		455 (61.2)		106 (14.2)			140 (18.9)			
	Nondaily	1279 (63.2)		106 (8.3)		761 (59.5)		172 (13.4)			240 (18.8)			
**Frequency of using e-cigarettes**														.26
	Noncurrent	202 (10)		13 (6.4)		129 (63.9)		32 (15.8)			28 (13.9)			
	Current	1821 (90)		125 (6.8)		1096 (60.2)		249 (13.7)			353 (19.4)			
**Device used for completing the experiment^g^**														<.001
	Mobile	1730 (85.5)		N/A^h^		1135 (65.6)		217 (12.5)			378 (21.8)			
	Desktop	293 (14.5)		N/A		177 (60.6)		84 (28.5)			32 (10.6)			

^a^The denominators for the percentages in these column are the n values from the “Full sample” column in the same row.

^b^Participants who responded “Other” or “Prefer not to say” to the biological sex question were excluded because of the small sample size (n=7).

^c^“Non-Hispanic other” includes non-Hispanic Asian, American Indian or Alaska Native, Native Hawaiian or Pacific Islander, multiracial, and other races.

^d^“Other orientations” status includes asexual, bisexual, gay, lesbian, pansexual, queer; questioning or unsure; another identity not listed; and prefer not to disclose.

^e^“Other” marital status includes widowed, separated, divorced, and other not specified in the available categories.

^f^“Other” employment status includes in military service, retired or disabled, and homemaker.

^g^Data for devices used to complete the experiment relate to those who decided to proceed to the Sticky experiment (n=1887).

^h^N/A: not applicable.

#### Eye-Tracking Data Processing and Completion Metrics

After reaching the recruitment goal, we used the software to generate various eye-tracking metrics (eg, fixations and dwell time), which took approximately 12 hours. Attention data were automatically streamed to the cloud as they were recorded, then processed for completeness while eye-tracking calibration quality was validated. The validation process measures eye-tracking data accuracy by how much the ground truth validation points differ from gaze predictions [25]. The validation results determine the usability of the data for each individual participant: a difference lower than a threshold value of 0.18 is considered usable [25].

Once the eye-tracking data were processed and validated, the software presented several experiment completion metrics that we used as the outcome of our study (Figure 1). Specifically, for data analysis, we generated a variable, *experiment completion,* to capture participants’ status of completing the experiment with four mutually exclusive outcomes: (1) did not initiate the experiment (ie, selected “I do not wish to proceed” just before the experimental portion), (2) initiated the experiment but did not complete it, (3) completed the experiment but did not generate usable data, and (4) completed the experiment and generated usable data. Completing the study and generating usable data were determined by the direct output from remote eye-tracking software. Experiment completion data were then merged with the Qualtrics survey data using unique participant IDs.

### Statistical Analysis

To understand which subgroups of young adult cigarette smokers were more likely to complete the remote eye-tracking study, we first assessed the sociodemographic backgrounds and tobacco use history of study participants as well as the outcomes from those completing the remote eye-tracking experiment. Next, we used Pearson chi-square tests to examine the bivariate associations between participant characteristics and the experiment completion variable. Post hoc analyses using the Fisher exact test [26] were further conducted to examine the subgroup differences, given the statistically significant chi-square test results. In addition, we ran separate logistic regression models to examine the associations between participant characteristics and experiment completion using models with various outcomes (initiated the experiment vs did not initiate the experiment, completed the experiment vs did not complete the experiment, and generated usable data vs generated nonusable data). After implementing the multivariable regression models to examine the associations between sociodemographic characteristics and experiment completion, we further controlled for participants’ tobacco use history, including frequency of cigarette smoking and current e-cigarette use. Finally, we controlled for the device (desktop vs mobile) used to complete the remote eye-tracking experiment. We analyzed the data using Stata (version 16.0; StataCorp LLC) and set the statistical significance to .05 (2-tailed).

### Ethics Approval, Participation, and Informed Consent

This study was approved by the institutional review board at Rutgers University (Pro2022000190). Participants who completed the survey (whether or not they completed the remote eye-tracking experiment) were compensated according to Qualtrics’ compensation policy. All participants viewed the informed consent page (including the details of the remote eye-tracking experiment) and agreed to participate in the survey study before they started the survey items.

## Results

Table 1 presents the distributions of sociodemographic backgrounds, tobacco use history, and experiment completion of the sample (N=2023). Approximately half of the participants (1018/2023, 50.3%) were female, and more than half (1284/2023, 63.47%) were non-Hispanic White. More than half of the participants (1279/2023, 63.22%) smoked cigarettes on a nondaily basis, and most of the participants (1821/2023, 90.01%) had used e-cigarettes at least once in the past 30 days. Of the 2023 participants, 1887 (93.28%) initiated the experiment, and 777 (38.41%) completed the experiment. Of the 777 participants who completed the experiment, 381 (49%) generated usable data. When considering only mutually exclusive categories, 6.72% (136/2023) of the participants did not initiate the experiment, 54.87% (1110/2023) initiated but did not complete the experiment, 19.57% (396/2023) completed the experiment but did not generate usable data, and 18.83% (381/2023) completed the experiment and generated usable data.

Table 1 shows that biological sex (*P*=.02), marital status (*P*=.003), and the electronic device used for completing the experiment (*P*<.001) were all significantly associated with remote eye-tracking experiment completion. Post hoc analyses show that female participants were more likely than male participants to generate usable data compared with generating nonusable data (*P*=.01). Among those who completed the experiment, participants who reported having a partner, being single, or having *other* marriage status were more likely to generate usable data (*P*=.002) compared with those who were married. In addition, participants who used mobile devices were more likely than those who used desktop devices to generate usable data compared with generating nonusable data (*P*<.001).

Multivariable logistic regressions (Multimedia Appendix 1) show that no participant characteristic was associated with initiating the experiment. In the multivariable regression model that controlled for all covariates, including electronic devices used for the eye-tracking experiment, the results show that non-Hispanic Black participants (adjusted odds ratio [AOR] 0.64, 95% CI 0.45-0.91) were less likely to complete the eye-tracking experiment than those who were non-Hispanic White and that those who lived with a partner (AOR 1.69, 95% CI 1.25-2.28) were more likely to complete the experiment than those who were married. In addition, female participants (AOR 1.46, 95% CI 1.01-2.11), those who currently used e-cigarettes (AOR 2.08, 95% CI 1.13- 3.82), and those who used mobile devices (AOR 5.10, 95% CI 3.05-8.52) were more likely to generate usable data than male participants, those who did not use e-cigarettes, and those who used desktop devices, respectively.

## Discussion

### Principal Findings

This is one of the first studies to provide a detailed description of the use of remote webcam-based eye-tracking technology to examine attention to tobacco marketing materials, particularly in the context of a web-based sample of participants recruited from a large-scale crowdsourcing effort. This is also one of the first studies to delineate the participant characteristics associated with completing remote eye-tracking experiments and generating usable data. Overall, 49% (381/777) of the young adult smoker participants who completed the remote eye-tracking experiment generated usable data within a 20-day data collection period. This equates to generating usable eye-tracking data from approximately 19 participants per day, potentially signifying a higher efficiency than traditional eye-tracking studies conducted in a laboratory setting. Although the total participation duration for the remote eye-tracking experiment was approximately 5 minutes, approximately 90 seconds of this time was devoted to eye-tracking data collection.

Our results show that several participant characteristics were associated with the completion of the eye-tracking experiment (eg, race and ethnicity) and with the generation of usable (vs nonusable) eye-tracking data (eg, biological sex, current e-cigarette use, and device type). The information gleaned from this study can be used to guide future remote eye-tracking research efforts to investigate attention to tobacco marketing stimuli among various populations.

### Remote Eye-Tracking Experiment Completion and Data Usability

Our study shows that of the 2023 participants who provided initial informed consent and started the web-based survey, very few participants (n=136, 6.72%) chose to opt out of the remote eye-tracking experiment even when all participants were informed that they needed to turn on their camera during the experiment and could still receive full compensation if they opted not to initiate the experiment. This finding suggests that, in general, our participants were open to participating in a remote eye-tracking task embedded in the web-based survey. In addition, according to our results, young adult cigarette smokers with various sociodemographic and tobacco use characteristics chose to initiate the experiment in a similar pattern, suggesting that receptivity may be high across various groups.

However, our results also show that more than half of the participants (1110/1887, 58.82%) initiated the remote eye-tracking experiment but did not complete it. There are several reasons why participants might not complete the experiment. Participants might have started the experiment but did not meet the technical requirements—such as having a camera installed on their electronic device—or they may have been unwilling or unable to turn on the camera at this point once it became mandatory. In the case of those who turned on their camera, they might have had issues related to low internet speed or a webcam with a low sample rate that generated unsatisfying video resolution. In addition, some of these participants may have intentionally closed the browser or timed out before reaching the end of the experiment. This could be due to concerns about personal privacy or because the participants’ environment prevented them from engaging in a webcam-based experiment at that time.

Furthermore, our results show that a portion of study participants (396/777, 51%) completed the experiment but did not generate usable eye-tracking data. This might be because of the lighting setup in the participants’ environment (background light that was too dark or too strong) or large head or body movements during the experiment. Other possible reasons for generating nonusable data include participants not paying attention to the stimuli during the experiment and not following the calibration dots. It has been shown that eye-tracking data accuracy is directly determined by whether participants can focus on the stimuli with their full attention [27]. In addition, reflections from the participants’ glasses may have interfered with the ability to collect eye-tracking gaze data.

### Characteristics of Participants by Experiment Completion and Data Usability

Our results further suggest that the majority of our participants (1613/1887, 85.48%) who initiated the eye-tracking experiment used mobile devices rather than desktop devices. This distribution may reflect the overall electronic device preferences of the crowdsourced participants recruited from the web-based study panels. Importantly, our analysis shows that although participants who used mobile devices and those who used desktop devices did not differ in their completion of the experiment, those who used mobile devices were approximately 5 times as likely to generate usable data compared with those who completed the experiment on their computers. We suspect that this may be because of the fewer technical requirements needed to achieve high-quality data collection through mobile devices compared with desktop devices. Alternatively, mobile device users may have different user preferences and backgrounds that contribute to this outcome; for example, mobile device users may be more attentive to the social media marketing stimuli used in our experiment, and therefore they generate more usable data. Future research is needed to explore different contributing factors that lead to generating usable (vs nonusable) eye-tracking data between mobile and desktop users (and the potential implications of this) to inform researchers of best practices for performing such experiments on both devices.

We also found that current e-cigarette users were more likely than nonusers to generate usable data. It is possible that current e-cigarette users were more likely to be interested in e-cigarette–related marketing materials and therefore paid more attention to the stimuli, which resulted in their generating more usable data than non–e-cigarette users. Further analysis based on the eye-tracking data collected from this study will be used to investigate the differences in the eye-tracking metrics (eg, fixations and dwell time) related to viewing the e-cigarette marketing stimuli between e-cigarette users and nonusers. In addition, we observed that female participants were more likely to generate usable data than male participants, even after controlling for all other potential contributing factors. As this is one of the first studies to explore the sociodemographic differences in participants’ completion of a remote eye-tracking experiment, little evidence is available to explain this difference among the sexes. In the same vein, little evidence is available to explain the demographic differences (race and ethnicity and marital status) that we observed in completion (vs noncompletion) of the remote eye-tracking experiment; for example, we observed that non-Hispanic Black participants were less likely to complete the experiment than non-Hispanic White participants. We suspect that participants’ real-time environment (eg, at work or home, in public or private spaces, with or without others in the background) and engagement in other activities (eg, childcare and performing chores) when completing the experiment may be relevant to study participants’ demographic characteristics. These factors may also lead to differential experiment completion outcomes because they may directly affect participants’ willingness to turn on their cameras or affect their ability to follow instructions or fully engage with the experiment.

More studies are needed to look into potential explanations of these findings and take into consideration the implications of the differential completion outcomes from participants with various demographic characteristics. If future remote eye-tracking studies continue to show variance in completing the experiment among participants of different sexes and races and ethnicities, it may be necessary for researchers to oversample certain subgroups in remote eye-tracking studies to achieve a balanced data set. This may be particularly true for race and ethnicity and reaching Black and other underrepresented participants, given that one of the potential benefits of remote eye-tracking methodology is the ability to reach and include a more diverse sample, which is important in nicotine and tobacco research for addressing known health disparities.

### Recommendations for Improving Experiment Completion and Data Usability

On the basis of our experience in implementing this study, we recommend several strategies to increase data completion and data usability rates for future remote eye-tracking studies. First, researchers can include certain technical requirements for participant eligibility, such as using high-speed internet and using a computer equipped with a high-speed camera. Second, researchers can add certain constraints regarding study completion in the informed consent form—such as participants needing to complete the remote webcam-based eye-tracking experiment, which requires turning on the camera—to receive the full compensation. This may discourage participants who do not wish to turn on their cameras from initiating the experiment and reduce the possibility of participants exiting the experiment before finishing. Third and last, researchers can show participants the instructions before or during the eye-tracking experiment, including reminding them to keep their heads still, avoid sitting near windows, and keep light sources above or in front of them rather than behind them. Useful instructions for study participants to follow are presented in Textbox 1.

**Tips to be included in remote eye-tracking study instructions to improve data usability.**

**Before we begin, here are a few tips to help you to better self-calibrate:**

- You must sit still and centered for the entire session.
- You must have a strong internet connection.
- Make sure you are in a well-lit room with no light or window behind you.
- Your computer should be placed on a steady table or desk.
- Make sure you are in a comfortable seated position.
- Make sure your sound is turned on.
- Please remove your glasses if you do not need them to see the screen clearly.
- If you need your glasses, ensure that you are in a well-lit room with no light source directly in front that can cause a glare.
- Before you start the session, ensure that you will have at least 5 minutes uninterrupted.

### Potential Challenges of Conducting Remote Eye-Tracking Experiments

Given the promising utility of the remote eye-tracking experiment, it is important to note the potential challenges that may arise before and during such an experiment within academic settings. First, although our overall data usability rate was low, almost half (381/777, 49%) of those who completed the experiment generated usable data. Future studies may need to factor in potential data loss when planning participant recruitment and adopt strategies introduced in this paper to improve data usability rates. Future studies may also consider how remote and in-person laboratory studies may be used in tandem; for example, research might incorporate a remote eye-tracking study into a survey with a large participant sample to explore general viewing patterns of stimuli of interest, which may then stimulate research questions and hypotheses that are examined in a more controlled in-person laboratory study. Second, there are limited opportunities for researchers to obtain raw eye-tracking data through using the software. Only some categories of eye-tracking metrics were provided by the software (eg, fixation and dwell time), limiting researchers’ ability to gain a more comprehensive understanding of the data generated from the experiments. Third, there may be challenges related to protecting participant privacy because remote eye-tracking data collection requires recording a video of the participant’s face. It is recommended that researchers review and evaluate the privacy policies of the eye-tracking software, including limitations on transferring participant data (eg, personal identifiers or video recordings) to researchers or third parties and permanently deleting the data after certain periods of time. It is recommended that researchers include these policies during their submission of the study protocol to an institutional review board. Fourth and last, according to our findings, certain characteristics of study participants who generated usable eye-tracking data may be different from those of study participants who did not generate usable data or did not complete the experiment. Therefore, the results generated from remote eye-tracking experiments may need to be interpreted with caution to avoid population bias.

### Study Limitations

The generalizability of our remote eye-tracking study may be limited because of the following reasons. First, the completion rates of the eye-tracking experiment among our participants—young adult cigarette smokers—may be different from those of similar studies among participants with different sociodemographic characteristics or tobacco use history. Specifically, young adults may be more skilled in successfully navigating the technical requirements (eg, turning on the camera) of remote eye-tracking software and less worried about web-based privacy issues than older adults. By contrast, older current cigarette smokers may be less likely to perform attention-intensive tasks such as repeatedly following the movements of dots and carefully viewing the assigned stimuli when they experience nicotine withdrawal symptoms [28]. This may be especially true when the eye-tracking experiment is embedded at the end of a survey after participants have already responded to a series of survey questions and when no additional compensation is provided to encourage the specific completion of the experiment. Second, it is possible that study participants who did not wish to take part in the remote eye-tracking experiment may not have agreed with the study consent to start with. Therefore, we were not able to assess the backgrounds and characteristics of this particular group. Third, the rates of completing the experiment and generating usable data may be different when the remote eye-tracking experiment is designed differently; for example, our experiment involves participants completing 2 calibration points and responding to a survey question at the end, which may be more complicated than other experiments that include 1 or no calibration points or no survey questions. Adding calibration points may improve the accuracy and usability of the data generated by the eye-tracking software but may also potentially extend the length of the experiment and reduce the experiment completion rate. Fourth and last, this study only introduced the process of designing 1 particular remote eye-tracking experiment using 1 particular software platform (ie, Sticky by Tobii). Experiments using other remote eye-tracking tools (eg, Webgazer.js) or with different study designs or experiment setups may involve unique challenges and generate different experiment completion outcomes [29].

### Conclusions

Eye-tracking methodology can greatly advance researchers’ and policy makers’ understanding of individuals’ visual engagement with marketing and communication materials in tobacco control research and tobacco regulatory science. Although in-person laboratory methods are considered the gold standard for collecting these types of data, recently developed remote webcam-based eye-tracking technology integrated with large-scale crowdsourcing efforts may be a particularly time-efficient and resource-effective tool for addressing limitations posed by in-person research. This paper describes the process of designing a remote eye-tracking study to explore young adults’ attention to e-cigarette social media marketing materials. The experiment completion outcome data from this study are promising because the study generates usable eye-tracking data from approximately 19 participants per day during a 20-day data collection period. The study also provides information on the characteristics of study participants who might be more likely to complete the remote eye-tracking experiment and generate usable eye-tracking data. We believe that remote eye-tracking experiments, integrated with large-scale crowdsourcing efforts, will be a useful tool for the tobacco regulatory science research community to expand the collection of eye-tracking data on a variety of tasks relevant to the impact of tobacco marketing.

## Appendix

Multimedia Appendix 1 Characteristics of participants (web-based sample of young adult cigarette smokers) and devices used for completing the remote eye-tracking experiment on a Qualtrics survey.

## Abbreviations

AOI: area of interest
AOR: adjusted odds ratio

## Data Availability

Survey data may be available upon request.
