# Supplementary material for: Examining Visual Attention to Tobacco Marketing Materials Among Young Adult Smokers: Protocol for a Remote Webcam-Based Eye-Tracking Experiment
Source: JMIR Res Protoc. 2023 Apr 13;12:e43512. doi: 10.2196/43512 (PMC10141307; doi:10.2196/43512)
Supplement: Multimedia Appendix 1 [file resprot_v12i1e43512_app1.docx]

**Table S1.** Characteristics of participants (online sample of young adult cigarette smokers) and devices used for completing the remote eye-tracking experiment on a Qualtrics survey (N=2023).

|  | | Initiated the experiment (base outcome: did not initiate), n=2016, AOR^a^ (95% CI) | | Completed the experiment (base outcome: did not complete the experiment), n=1880, AOR (95% CI) | | | Generated usable data (base outcome: generated nonusable data), n=661, AOR (95% CI) | | |
| --- | --- | --- | --- | --- | --- | --- | --- | --- | --- |
|  | | **Model A** | **Model B** | **Model A** | **Model B** | **Model C** | **Model A** | **Model B** | **Model C** |
| **Biological sex^b^** | | | | | | | | | |
|  | Female | 1.10 (0.74-1.61) | 1.07 (0.73-1.58) | 0.86 (0.70-1.06) | 0.86 (0.70-1.07) | 0.89 (0.72-1.10) | *1.53 (1.07-2.18)* | *1.58 (1.10-2.25)* | *1.46 (1.01-2.11)* |
|  | Male | Reference | Reference | Reference | Reference | Reference | Reference | Reference | Reference |
| **Age (years)** | | | | | | | | | |
|  | 18 to 21 | Reference | Reference | Reference | Reference | Reference | Reference | Reference | Reference |
|  | 22 to 25 | 1.24 (0.66-2.33) | 1.20 (0.65-2.32) | 0.86 (0.59-1.26) | 0.86 (0.58-1.26) | 0.86 (0.58-1.26) | 0.58 (0.30-1.15) | 0.59 (0.30-1.16) | 0.65 (0.32-1.33) |
|  | 26 to 29 | 1.90 (0.98-3.77) | 1.87 (0.98-3.59) | 0.94 (0.65-1.36) | 0.94 (0.64-1.38) | 0.95 (0.65-1.38) | 0.58 (0.30-1.12) | 0.59 (0.30-1.16) | 0.62 (0.31-1.23) |
|  | 30 to 34 | 1.75 (0.91-3.37) | 1.70 (0.87-3.32) | 0.91 (0.62-1.34) | 0.92 (0.61-1.37) | 0.93 (0.62-1.38) | 0.55 (0.27-1.10) | 0.56 (0.27-1.15) | 0.58 (0.28-1.21) |
| **Race/ethnicity** | | | | | | | | | |
|  | Non-Hispanic White | Reference | Reference | Reference | Reference | Reference | Reference | Reference | Reference |
|  | Non-Hispanic Black | 1.01 (0.53-1.92) | 1.07 (0.60-2.05) | *0.63 (0.45-0.90)* | *0.64 (0.45-0.91)* | *0.64 (0.45-0.91)* | 1.73 (0.89-3.37) | 1.93 (0.98-3.78) | 2.00 (0.97-3.90) |
|  | Hispanic | 0.82 (0.52-1.30) | 0.85 (0.53-1.35) | 0.99 (0.77-1.29) | 0.99 (0.76-1.28) | 0.98 (0.76-1.28) | 1.00 (0.64-1.55) | 1.00 (0.64-1.55) | 1.06 (0.66-1.68) |
|  | Non-Hispanic other^c^ | 0.59 (0.33-1.06) | 0.62 (0.35-1.12) | 0.91 (0.63-1.31) | 0.90 (0.62-1.31) | 0.90 (0.62-1.30) | 1.15 (0.62-2.13) | 1.19 (0.64-2.23) | 1.52 (0.79-2.95) |
| **Sexual orientation** | | | | | | | | | |
|  | Straight | Reference | Reference | Reference | Reference | Reference | Reference | Reference | Reference |
|  | Nonstraight^d^ | 0.85 (0.57-1.29) | 0.87 (0.57-1.30) | 1.03 (0.82-1.29) | 1.03 (0.81-1.29) | 1.03 (0.82-1.29) | 0.82 (0.55-1.21) | 0.80 (0.54-1.19) | 0.74 (0.49-1.11) |
| **Marital status** | | | | | | | | | |
|  | Legally married | Reference | Reference | Reference | Reference | Reference | Reference | Reference | Reference |
|  | Living with partner | 1.63 (0.94-2.83) | 1.61 (0.92-2.78) | *1.63 (1.21-2.20)* | *1.62 (1.21-2.19)* | *1.69 (1.25-2.28)* | *1.77 (1.08-2.91)* | *1.70 (1.03-2.81)* | 1.48 (0.88-2.49) |
|  | Single, never married | 1.37 (0.86-2.19) | 1.38 (0.86-2.19) | 1.09 (0.83-1.43) | 1.09 (0.82-1.43) | 1.11 (0.85-1.47) | *1.70 (1.06-2.74)* | *1.65 (1.02-2.66)* | 1.56 (0.95-2.58) |
|  | Other^e^ | 1.48 (0.60-3.69) | 1.44 (0.57-3.59) | 1.30 (0.81-2.09) | 1.31 (0.81-2.11) | 1.39 (0.86-2.24) | *2.77 (1.22-6.27)* | *2.74 (1.20-6.23)* | 2.07 (0.90-4.78) |
| **Education** | | | | | | | | | |
|  | Less than high school | Reference | Reference | Reference | Reference | Reference | Reference | Reference | Reference |
|  | High school | 1.01 (0.47-2.17) | 1.03 (0.47-2.19) | 1.00 (0.67-1.50) | 0.9 (0.66-1.49) | 0.98 (0.65-1.46) | 0.97 (0.49-1.92) | 0.86 (0.43-1.72) | 0.83 (0.41-1.69) |
|  | Less than college degree | 0.88 (0.40-1.93) | 0.91 (0.42-2.01) | 0.96 (0.63-1.45) | 0.95 (0.62-1.44) | 0.92 (0.61-1.40) | 1.13 (0.56-2.28) | 0.98 (0.48-2.01) | 1.03 (0.49-2.14) |
|  | College degree or higher | 0.87 (0.37-2.08) | 0.91 (0.38-2.20) | 1.25 (0.78-2.00) | 1.22 (0.76-1.97) | 1.14 (0.70-1.85) | 0.75 (0.34-1.65) | 0.64 (0.28-1.46) | 0.89 (0.38-2.08) |
| **Current employment status** | | | | | | | | | |
|  | Work full time | Reference | Reference | Reference | Reference | Reference | Reference | Reference | Reference |
|  | Work part time | 1.65 (0.90-3.02) | 1.66 (0.91-3.05) | 1.05 (0.78-1.41) | 1.05 (0.78-1.41) | 1.06 (0.79-1.42) | 1.00 (0.60-1.65) | 1.07 (0.64-1.77) | 1.17 (0.69-1.98) |
|  | Unemployed | 1.16 (0.69-1.95) | 1.15 (0.69-1.96) | 1.31 (0.99-1.74) | 1.32 (0.99-1.74) | 1.31 (0.99-1.74) | 0.97 (0.61-1.52) | 1.00 (0.63-1.59) | 1.11 (0.69-1.78) |
|  | Other^f^ | 1.40 (0.72-2.70) | 1.40 (0.72-2.71) | 1.33 (0.96-1.86) | 1.34 (0.96-1.87) | 1.36 (0.97-1.89) | 1.55 (0.89-2.70) | 1.63 (0.93-2.83) | 1.53 (0.86-2.71) |
| **Currently in school** | | | | | | | | | |
|  | No | Reference | Reference | Reference | Reference | Reference | Reference | Reference | Reference |
|  | Yes | 1.13 (0.73-1.75) | 1.15 (0.73-1.76) | 1.02 (0.80-1.30) | 1.02 (0.80-1.30) | 1.01 (0.79-1.28) | 1.00 (0.67-1.51) | 1.02 (0.67-1.54) | 1.10 (0.71-1.69) |
| **Subjective financial status** | | | | | | | | | |
|  | Comfortable | Reference | Reference | Reference | Reference | Reference | Reference | Reference | Reference |
|  | Meet needs | 0.74 (0.47-1.18) | 0.76 (0.48-1.21) | 1.11 (0.85-1.45) | 1.11 (0.85-1.44) | 1.11 (0.85-1.44) | 1.08 (0.69-1.69) | 1.06 (0.68-1.66) | 1.09 (0.68-1.75) |
|  | Just meet needs | 0.92 (0.56-1.53) | 0.91 (0.55-1.52) | 1.13 (0.86-1.48) | 1.12 (0.86-1.47) | 1.13 (0.86-1.48) | 0.88 (0.56-1.39) | 0.84 (0.52-1.33) | 0.76 (0.47-1.22) |
|  | Do not meet needs | 0.85 (0.45-1.60) | 0.86 (0.46-1.64) | 0.94 (0.67-1.32) | 0.94 (0.67-1.32) | 0.96 (0.68-1.34) | 0.69 (0.39-1.21) | 0.68 (0.39-1.19) | 0.58 (0.33-1.04) |
| **Cigarette smoking frequency** | | | | | | | | | |
|  | Nondaily | N/A^g^ | Reference | N/A | Reference | Reference | N/A | Reference | Reference |
|  | Daily | N/A | 1.31 (0.91-1.91) | N/A | 0.97 (0.78-1.19) | 0.98 (0.79-1.21) | N/A | 0.99 (0.69-1.41) | 0.93 (0.64-1.34) |
| **Current e-cigarette use** | | | | | | | | | |
|  | Noncurrent | N/A | Reference | N/A | Reference | Reference | N/A | Reference | Reference |
|  | Current | N/A | 1.05 (0.56-1.95) | N/A | 1.12 (0.80-1.58) | 1.11 (0.79-1.55) | N/A | *1.98 (1.10-3.56)* | *2.08 (1.13-3.82)* |
| **Device used for completing the experiment^h^** | | | | | | | | | |
|  | Screen | N/A | N/A | N/A | N/A | Reference | N/A | N/A | Reference |
|  | Mobile | N/A | N/A | N/A | N/A | 0.76 (0.57-1.02) | N/A | N/A | *5.10 (3.05-8.52)* |

^a^AOR: adjusted odds ratio.

^b^Participants who responded “Other” or “Prefer not to say” to the biological sex question were dropped from the regression models because of the small sample size (n=7).

^c^“Non-Hispanic other” includes non-Hispanic Asian, American Indian or Alaska Native, Native Hawaiian or Pacific Islander, multiracial, and other races.

^d^Nonstraight sexual orientation includes asexual, bisexual, gay, lesbian, pansexual, queer, questioning or unsure, another identity not listed, and prefer not to disclose.

^e^“Other” marital status includes widowed, separated, divorced, and other not specified in the available categories.

^f^“Other” employment includes in military service, retired or disabled, and homemaker.

^g^N/A: not available.

^h^Data for devices used to complete the experiment relate to those who decided to proceed to the Sticky experiment (n=1887).
